# History of the World Allergy Organization: ICACI XI, London 1982, Planning and Results

**DOI:** 10.1097/WOX.0b013e3182215644

**Published:** 2011-06-15

**Authors:** Robert J Davies

**Affiliations:** 1 96 Vanbrugh Park, Blackheath, London, SE3 7AL UK

## Abstract

*History of the World Allergy Organization: In 1951, the leaders in allergy from all over the world came together to form the International Association of Allergology and Clinical Immunology (IAACI). For the next 60 years, the allergy world converged at the IAACI triennial meetings, which became biennial in 2003. The international meetings, originally named the International Congress of Allergology and Clinical Immunology (ICACI), are now the World Allergy Congress (WAC) hosted by the World Allergy Organization (WAO). Everyone who has aspired to have worldwide recognition has played a part in IAACI-WAO. The History of the World Allergy Organization traces the global arc of the allergy field over the past 60 years*.

*The current officers of WAO elected to focus on this rich history, inviting prominent leaders who are interested in being part of this history project to write about their time with IAACI-WAO. This series will be presented in Cancún, México as part of the XXII World Allergy Congress (December 4-8, 2011). Leading up to the Congress in Cancún, the World Allergy Organization Journal is presenting segments of the History as part of the "Notes of Allergy Watchers Series," starting with this issue. Please enjoy*.

--*Michael A. Kaliner, MD*

Historian, and Past-President (2006-2007)

World Allergy Organization

## 

The commencement in 1979 of the planning and organization of the XI International Congress of Allergology and Clinical Immunology (ICACI) marks the exact midpoint between the first Congress in 1949 and the most recent in 2009, and highlights how far our organization and specialty have advanced in the last 30 years.

My first association with the IAACI was when I was asked by Jack Pepys to be Co-Chairman of the London Congress. At the time, I was Secretary, and then became President, of the British Society for Allergy and Clinical Immunology. Our first major task was to book a suitable venue in London for this triennial meeting for approximately 2,000 delegates. A bold decision was made to book the Barbican Centre for Arts and Conferences, which was still under construction. The Organizing Committee watched with increasing anxiety as development of this centrally located but complex structure proceeded, and were delighted and relieved to be invited to the opening concert, attended by Her Majesty Queen Elizabeth II, barely weeks before the Congress began!

The Congress was held 33 years after the inauguration of the IAACI, an important landmark highlighted in a plenary lecture by Max Samter entitled "Allergy 33 Years On". The IAACI President was Dr. Carl Arbesman, whose program message to the Congress emphasized the difficult times the specialty had been through, and he thanked the many present who not only had attended every Congress but also had kept the specialty alive. Sadly, Dr. Arbesman died just before the Congress started. The acting President, Dr. Alain De Weck, took his place. At this time in the development of the IAACI, it was mandatory to have the program and the main plenary sessions translated into Spanish, German, and French.

In our introduction to the scientific program, Jack and I stressed the importance of the continuing development of the basic immunopathologic knowledge underlying the advances in clinical practice. The key scientific and clinical areas covered in the 1982 program were--perhaps largely as they remain today--mediators of allergic tissue reactions, the influence of antenatal and neonatal factors, the development of antiallergic drugs, regulation of the IgE response, clinical and experimental implications of long-term IgE and short-term IgG-STS antibodies. Occupational respiratory allergy was a major theme, but this is one area where increased recognition of occupational allergens and successful prevention strategies have helped to reduce disease burden and the need to focus on this as a major congress topic (see **Photo, Supplemental Digital Content 1**, http://links.lww.com/WAOJOURNAL/A2).

Approximately 900 abstracts were received for the London Congress, and more than 2,000 delegates attended the meeting. These were the highest numbers in Congress history to that date (see **Photo, Supplemental Digital Content 2**, http://links.lww.com/WAOJOURNAL/A3).

Allergy in those days continued very much as a close-knit family of researchers and clinicians, exemplified by the chairs of the main plenary symposia. These included Bram Rose, Jacques Charpin, Albert Oehling, Frank Austen, Max Samter, Terumasa Miyamoto, Larry Lichtenstein, Felicidad Cua-Lim, Francois-Bernard Michel, Lee Frick, Brita Stenius-Aarnalia, Bill Frankland, Freddie Hargreave, and Barry Kay. Not only were the major researchers and clinicians of their day present at the Congress but also many of those who would become internationally recognized in the future, who had been fellows of the early allergy pioneers. My own connections were as a research fellow with Jack Pepys in London, and then with John Salvaggio in New Orleans.

The Patron of the Congress was Her Majesty Queen Elizabeth II, and the social events began in typical British fashion with the marching band of the Grenadier Guards, who also performed at the Congress concert in the beautiful Barbican Hall in the middle of the week. The faculty dinner was held in the Great Hall of St. Bartholomew's Hospital, attended by His Royal Highness the Duke of Gloucester. Bart's Hospital, the oldest in the United Kingdom, dating from the 11th century, was famous as the place where in the 17th century William Harvey, the Physician in Charge, described the circulation of the blood. A plenary congress session was held at the hospital every day, giving congress delegates the opportunity to visit this important historical venue.

The London Congress was a financial success, and as a result of my involvement with this successful event, I was invited to become Treasurer of the Organization. Before 1982, the financial benefits to IAACI of organizing the Congress were variable, and the expenditure for each 3-year term depended on the profit, if any, made by the previous Congress. I took the opportunity, as the newly elected Treasurer, of instigating a levy of 12% of the Congress fee to be paid to the Organization, independent of any profits realized, to ensure a steady income to fund the continuing development of the IAACI. I remained as Treasurer for 3 terms, passing the baton to Mike Kaliner, and then moved to the posts of 3rd and 2nd Vice President, serving a total of 19 years on the World Allergy Organization Board, right up until the Strategic Planning meeting in Stockholm, when the IAACI became the World Allergy Organization. Throughout this time the Congresses became increasingly successful both scientifically and financially.

I had the pleasure of being involved with the development, in 1996, of the World Allergy Forum, and of serving as the first Chairman. We were concerned that all the IAACI's educational programs were conducted on a one-off basis, with no long-term continuity, and we hoped to secure long-term funding for a premium program that would become a regular feature of our regional societies' programming. With the commitment of funding from Fisons (one of the companies which, after a series of mergers, evolved into Novartis), and the enthusiastic support of Keith Allan and Ken Rainey, the World Allergy Forum was created at a meeting in London, and has gone on to become the longest running educational activity of the World Allergy Organization.

## Note

The author was Board Member, Treasurer, Vice President, and Co-Chair of the XI International Congress of Allergology and Clinical Immunology, London, 17-22 October, 1982.

Supplemental digital content is available for this article. Direct URL citations appear in the printed text and are provided in the HTML and PDF versions of the journal's Web site (http://www.waojournal.org).

